# Biocatalysis as a green route for recycling the recalcitrant plastic polyethylene terephthalate

**DOI:** 10.1111/1751-7915.12714

**Published:** 2017-04-12

**Authors:** Ren Wei, Wolfgang Zimmermann

**Affiliations:** ^1^ Department of Microbiology and Bioprocess Technology Institute of Biochemistry Leipzig University Johannisallee 21‐23 04103 Leipzig Germany

## Abstract

Biocatalysis can enable a closed‐loop recycling of post‐consumer PET waste.

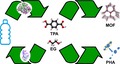

## Introduction

The global production of fossil‐based plastics has grown more than 20‐fold since 1964 to 322 million tons in 2015, and a slowdown of this rate is not expected (Ellen MacArthur Foundation and World Economic Forum, 2014; PlasticsEurope, 2016). Many materials derived from synthetic polymers have already replaced their natural counterparts in all areas of human life. The majority of plastics are short‐lived products which are disposed within 1 year after manufacture. However, only 14% of plastic packaging materials used worldwide is currently collected for recycling while another 14% is incinerated for energy recovery (Ellen MacArthur Foundation and World Economic Forum, 2014). The remaining 72% of plastic packaging is not recovered with 40% land filled and 32% estimated to completely escape the collection system. This part of plastic waste ends up in diverse natural habitats, especially in oceans where it can cause serious environmental damages (Andrady, 2015; Jambeck *et al*., 2015). Therefore, innovative technologies to improve the recycling of plastics and to reduce the consumption of non‐renewable fossil feed stocks are required.

Polyethylene terephthalate (PET) is the most widely used synthetic polyester. It is a thermoplastic of high‐molecular‐weight composed of terephthalic acid (TPA) and ethylene glycol (EG). PET can exist as both an amorphous and a semi‐crystalline polymer (Webb *et al*., 2013). Owing to its excellent physical and chemical properties, PET finds numerous applications as textile fibres, packaging materials and beverage bottles. PET is generally referred to as ′polyester′ in the textile industry which consumes the majority of the PET produced globally. In 2014, 49.2 million tons of PET fibres was produced worldwide (Fiber Economics Bureau, 2015). In 2015, the global production of PET resins was 27.8 million tons which was dominantly used for the manufacture of packaging materials and beverage bottles (Plastic Insight, 2016). Almost half of the postconsumer PET bottles worldwide are collected for mechanical recycling to produce polyester fibres (Ellen MacArthur Foundation and World Economic Forum, 2014).

Polyethylene terephthalate made from renewable biomass (bio‐PET) is becoming of industrial interest lately. EG derived from sugarcane ethanol (Tsiropoulos *et al*., 2015) and TPA derived from sugar beet paraxylene (Collias *et al*., 2014; Smith, 2015) can be utilized to replace their fossil‐based counterparts to produce bottle‐grade PET. Although fossil feedstocks can be saved by the commercialization of bio‐PET bottles, the challenges for their recycling remain as their recalcitrant properties are the same as those of petroleum‐derived PET bottles (Chen *et al*., 2016).

## Microbial polyester hydrolases can degrade PET

Polyethylene terephthalate has been previously considered as recalcitrant to biological degradation. However, fungal and bacterial polyester hydrolases exhibiting hydrolytic activity against PET films and fibres have been reported recently (Zimmermann and Billig, 2011; Chen *et al*., 2013; Wei *et al*., 2014c; Wei and Zimmermann, 2017) (Table 1). Among the reported microbial polyester hydrolases, cutinases and their homologues have shown the greatest potential for PET hydrolysis (Müller *et al*., 2005; Ronkvist *et al*., 2009; Herrero Acero *et al*., 2011; Wei *et al*., 2014c). Cutinases are enzymes capable of hydrolysing cutin, an aliphatic polyester found in the plant cuticle (Kolattukudy, 1981; Heredia, 2003). Müller *et al*. (2005) first reported that low‐crystalline (lc) PET film with 10% crystallinity prepared by melt pressing of PET beverage bottles could be degraded by the polyester hydrolase TfH from *Thermobifida fusca* DSM43793 at 55°C. However, a reaction time of 3 weeks with an exchange of the enzyme solution every week was required to achieve a weight loss of approximately 50% of the PET film. Ronkvist *et al*. (2009) reported a 97 ± 3% weight loss of an lc PET film with 7% crystallinity within only 96 h of reaction at 70°C with the fungal cutinase HiC from *Thermomyces* (formerly *Humicola*) *insolens*. A comparable degradation performance with amorphous PET materials has been also demonstrated with LC‐cutinase at 70°C (Sulaiman *et al*., 2014), a polyester hydrolase homologous to TfH identified by a metagenomics approach (Sulaiman *et al*., 2012), and by a variant of TfCut2 at 65°C, derived from *T. fusca* KW3 differing only in two amino acid residues from TfH (Wei *et al*., 2016).

**Table 1:** Microbial polyester hydrolases reported to cause significant weight losses of PET materials

Enzyme	Source	Reaction conditions		Weight loss	Crystallinity of PET	PET source and preparation	References
		Temperature	Time				
TfH	*Thermobifida fusca* DSM43793	55°C	3 weeks	≈ 50%	10%	Melt pressing of a beverage bottle (Granini AG, Nieder‐Olm, Germany)	(Müller *et al*., 2005)
TfH	*Thermobifida fusca* DSM43793	55‐65°C	48 h	≤ 14%	Not reported	Amorphous PET film (Goodfellow GmbH, Bad Nauheim, Germany)	(Then *et al*., 2015)
BTA‐2				≤ 4%			
Tfu_0882	*Thermobifida fusca* YX	55‐65°C	48 h	≤ 5%			(Then *et al*., 2015)
TfCut1	*Thermobifida fusca* KW3	55‐65°C	48 h	≤ 11%			(Then *et al*., 2015)
TfCut2				≤ 12%			
Variants of TfCut2	*Thermobifida fusca* KW3	65‐80°C	48 h	≤ 25%			(Then *et al*., 2015, 2016)
		65°C	50 h	≤ 45%			(Wei *et al*., 2016)
A double mutant of Cut190	*Saccharomonospora viridis* AHK190	63°C	3 days	13.5 ± 0.5%	Not reported	Amorphous PET film (Goodfellow Cambridge, Ltd., Tokyo, Japan)	(Kawai *et al*., 2014)
				27 ± 1%		Amorphous PET film (Sanwa Supply Inc., Okayama, Japan)	
LC‐cutinase	Metagenome from plant compost	50‐70°C	24 h	≤ 25%	Not reported	Amorphous PET film (Sanwa Supply Inc., Okayama, Japan)	(Sulaiman *et al*., 2014)
HiC	*Thermomyces* (formerly *Humicola*) *insolens*	70°C	96 h	97 ± 3%	7%	Amorphous PET film (Goodfellow Cambridge, Ltd.)	(Ronkvist *et al*., 2009)

**Figure 1:**
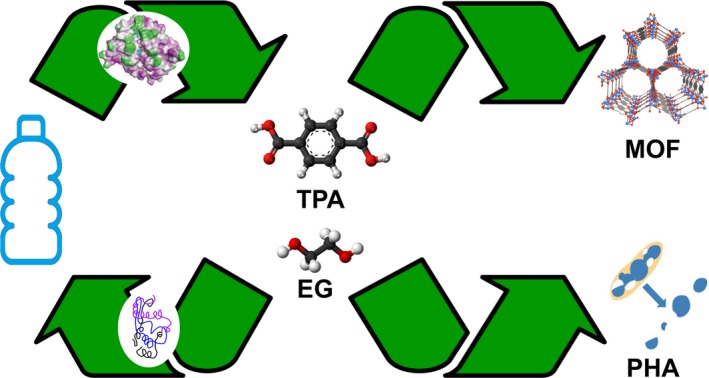
Biocatalysis for a closed‐loop recycling of postconsumer PET. TPA, terephthalic acid; EG, ethylene glycol; MOF, metal‐organic framework; PHA, polyhydroxyalkanoate.

Microbial polyester hydrolases can enable a complete depolymerization of lc PET into its monomers TPA and EG that can be reused for the synthesis of virgin PET (Fig. 1). Recycled TPA can also be employed for the synthesis of products with significantly added value such as metal‐organic frameworks (MOF) (Manju *et al*., 2013). Enzymatically recycled PET monomers may also become substrates for *Pseudomonas putida* cell factories to produce biodegradable, value‐added bioplastics such as polyhydroxyalkanoates (PHA) (Wierckx *et al*., 2015).

The PET hydrolases Thc_cut1 from *T. cellulosilytica* (Pellis *et al*., 2016a) and HiC (Hunsen *et al*., 2008a,b) have been shown to efficiently synthesize aliphatic polyesters as well as to catalyse a ring‐opening polymerization of lactones. These enzymes could therefore be also employed for the synthesis of polyesters from recycled monomers and open up avenues for novel closed‐loop recycling technologies of postconsumer PET waste based on biocatalysis (Fig. 1).

Chemical methods applied for the depolymerization of PET require high temperatures and the use of toxic chemicals (Awaja and Pavel, 2005). In contrast, a biocatalytic degradation of PET is providing a green technology performed at mild temperature and pH conditions in the absence of hazardous chemicals (Wei and Zimmermann, 2017). PET hydrolases show a high specificity for the ester bonds in PET. Hence, enzymatic degradation of PET in composite materials such as PET‐polyethylene blends, metallized PET‐based packaging films or in textiles containing PET could open up further opportunities to recover value from plastic waste using a biocatalytic approach (Gallagher, 2003).

## Key challenges for biocatalytic PET recycling

The efficient enzymatic hydrolysis of PET by polyester hydrolases still presents a number of challenges for the further development of a biocatalytic PET waste recycling technology. The presence of the aromatic building block TPA in PET provides stiffness to the polymer chain and is a major reason for its low biodegradability (Marten *et al*., 2003, 2005). As a result, PET exhibits a glass transition temperature (*T*_g_) of above 70°C and a melting point (*T*_m_) of over 230°C (Alves *et al*., 2002). This prohibits the application of enzymes at the *T*_m_ of PET. However, at its *T*_g_, the amorphous regions of PET become more flexible and accessible to enzymatic attack. Indeed, enzymatic hydrolysis performed at a reaction temperature close to its *T*_g_ resulted in an efficient degradation of lc PET films (Ronkvist *et al*., 2009). As a consequence, polyester hydrolases for the degradation of PET need to exhibit thermal stability properties at elevated temperatures of ≥ 70°C.

Computational simulation using structural models of polyester hydrolases from *Thermomonospora curvata* DSM43183 revealed that their flexible catalytic triad is more prone to unfold at high reaction temperatures near *T*_g_ of PET (Wei *et al*., 2014b). This unfolding process can be suppressed when Ca^2+^ or Mg^2+^ bind to the potential metal ion binding site of TfCut2, a homologous polyester hydrolase (Then *et al*., 2015). Indeed, the thermal stability and hydrolytic activity against PET of polyester hydrolases from *T. alba AHK119* (Thumarat *et al*., 2012), *T. fusca* KW3, YX and DSM43793 (Then *et al*., 2015) as well as from *Saccharomonospora viridis* AHK190 (Kawai *et al*., 2014) showed a positive correlation with the presence of Ca^2+^ and Mg^2+^ in the reaction medium. The replacement of the metal ion binding site with a disulfide bridge allowed the hydrolysis of PET films by TfCut2 variants at reaction temperatures up to 80°C independent of Ca^2+^ (Then *et al*., 2016). Phosphate anions also had a stabilizing effect on bacterial polyester hydrolases and increased their PET degradation performance (Schmidt *et al*., 2016).

Among the bacterial polyester hydrolases, the metagenome‐derived LC‐cutinase has shown an outstanding thermal stability at 70°C and superior hydrolytic activity against lc PET (Sulaiman *et al*., 2014). Thus, the microbial biodiversity in environmental samples may allow the identification of further promising polyester hydrolases by metagenomic approaches. Moreover, protein engineering focusing on key amino acid residues involved in the thermostabilization of polyester hydrolases can be expected to provide further biocatalysts as candidates for industrial PET recycling processes. For these purposes, the availability of high‐throughput screening methods plays an important role for the rapid detection of novel PET‐hydrolysing enzymes from the environment and their variants created by genetic engineering (Wei *et al*., 2012).

Recently, a polyester hydrolase exhibiting PET‐hydrolysing activity at 30°C was identified from *Ideonella sakaiensis* 201‐F6, isolated from a PET bottle recycling site (Yoshida *et al*., 2016). However, due to the inertness of PET at this temperature as a result of its intrinsic polymer properties, a biocatalytic recycling at low reaction temperatures is unlikely to be feasible.

The enzymatic hydrolysis of PET is a surface erosion process (Müller, 2006). The interaction of the biocatalyst with the polymeric substrate therefore plays an essential role for an efficient hydrolysis performance. Crystal structural analysis of several bacterial polyester hydrolases revealed the location of the catalytic triad in a surface groove surrounded by several hydrophobic amino acids (Kitadokoro *et al*., 2012; Roth *et al*., 2014; Sulaiman *et al*., 2014). This structural feature has been considered as crucial for the accommodation of a large polymeric substrate with hydrophobic properties such as PET (Herrero Acero *et al*., 2011; Kitadokoro *et al*., 2012). The adsorption of the polyester hydrolases to the polyester surface could be augmented by the presence of exogenous binding modules. The fusion of the polymer binding module from a PHA‐hydrolysing enzyme or of fungal hydrophobins to Thc_cut1 resulted in a significantly enhanced adsorption and hydrolytic activity on the surface of PET films (Ribitsch *et al*., 2013, 2015).

Bis(2‐hydroxyethyl) terephthalate (BHET) and mono‐(2‐hydroxyethyl) terephthalate (MHET) are low‐molecular esters of TPA and EG that are released during the enzymatic degradation of PET (Vertommen *et al*., 2005; Wei *et al*., 2012). These intermediate hydrolysis products have been shown to inhibit the activity of the polyester hydrolases TfCut2 and LC‐cutinase (Barth *et al*., 2015a, 2016). MHET has a high affinity to the substrate binding pocket of TfCut2 and is hydrolysed at a very low rate. This inhibitory effect could be efficiently reduced using an ultrafiltration membrane reactor (Barth *et al*., 2015b). Thereby, inhibitory hydrolysis products did not accumulate in the enzyme reactor and could be easily collected together with TPA and EG. In another strategy to remove inhibitory intermediate hydrolysis products, the immobilized carboxylesterase TfCa from *T. fusca* KW3 (Billig *et al*., 2010; Oeser *et al*., 2010) with high activity against low‐molecular PET oligomers was employed together with the polyester hydrolases TfCut2 or LC‐cutinase in an enzyme reactor to hydrolyse lc PET films (Barth *et al*., 2016). As a result, an up to twofold increased yield in degradation products could be obtained. Similarly, lipase CalB also facilitated the degradation of PET catalysed by the fungal polyester hydrolase HiC (Carniel *et al*., 2016). A 7.7‐fold increase in the yield of TPA was obtained due to the removal of MHET hydrolysed by CalB. By these dual‐enzyme systems, PET monomers can be obtained in a one‐pot process in high purity simplifying their downstream processing.

The product inhibition of the polyester hydrolase TfCut2 by MHET could also be relieved by a single site substitution of a key amino acid residue involved in the interaction with an oligomeric PET model compound (Wei *et al*., 2016). A 2.7‐fold higher weight loss of lc PET films could be obtained with this enzyme variant than with the wild type TfCut2.

Beverage bottles and fibres with a high degree of crystallinity of above 30% represent the most abundant types of postconsumer PET materials and can presently not be efficiently hydrolysed in an enzymatic process (Wei and Zimmermann, 2017). Compared to a biaxially oriented PET film with 35% crystallinity, a lc PET film with 7% crystallinity was hydrolysed by HiC at a 25‐fold higher rate at 70°C (Ronkvist *et al*., 2009). While an approximately 42% weight loss of lc PET film was obtained with a TfCut2 variant within 50 h at 65°C, PET fibres with a higher degree of crystallinity of above 30% were only partially hydrolysed (Wei *et al*., 2016). Bottle‐grade PET also showed a low enzymatic degradability. About two orders of magnitude lower amounts of degradation products were released from this material compared to from amorphous PET with a degree of crystallinity of 1.9% at the same reaction conditions (Yoshida *et al*., 2016).

A mechanical pretreatment of postconsumer PET materials may also be necessary to enable a complete enzymatic hydrolysis by enlarging the available surface area for access to the enzymes. Reducing various PET materials to particles with a diameter of about 100 nm significantly increased their degradation by TfCut2 (Wei *et al*., 2014a). The Thc_cut1 catalysed degradation of lc PET powders also released higher amounts of degradation products than from PET films at the same reaction condition as a result of the increased surface area (Pellis *et al*., 2016b). PET particle sizes between 0.25 and 0.5 mm obtained by micronization of different postconsumer beverage bottles were shown to be more susceptible to an enzymatic hydrolysis by Thc_cut1 as a result of the increased accessible surface area for the enzyme (Gamerith *et al*., 2017).

## Concluding remarks

Microbial polyester hydrolases have shown their potential in the biocatalytic depolymerization of PET. For an efficient degradation of postconsumer PET plastic waste in an industrial process, the performance of the enzymes still requires substantial improvements. The discovery and engineering of novel polyester hydrolases exhibiting specific catalytic properties towards high crystalline and bottle‐grade PET materials therefore remain key challenges. This can be achieved by applying microbial biotechnology methodologies for identifying novel enzymes from the environment exploiting microbial biodiversity and by generating powerful variants by protein engineering using rational design and enzyme evolution strategies. If successful, these enzymes can make an important contribution towards a future sustainable closed‐loop plastic recycling industry.

## Conflict of interest

None declared.

## References

[mbt212714-bib-0001] Alves, N.M. , Mano, J.F. , Balaguer, E. , Meseguer Duenas, J.M. , and Gomez Ribelles, J.L. (2002) Glass transition and structural relaxation in semi‐crystalline poly(ethylene terephthalate): a DSC study. Polymer 43: 4111–4122.

[mbt212714-bib-0002] Andrady, A.L. (2015) Plastics in the oceans In Plastics and Environmental Sustainability. Hoboken, New Jersey, USA: John Wiley & Sons, Inc, pp. 295–318.

[mbt212714-bib-0003] Awaja, F. , and Pavel, D. (2005) Recycling of PET. Eur Polym J 41: 1453–1477.

[mbt212714-bib-0004] Barth, M. , Oeser, T. , Wei, R. , Then, J. , Schmidt, J. , and Zimmermann, W. (2015a) Effect of hydrolysis products on the enzymatic degradation of polyethylene terephthalate nanoparticles by a polyester hydrolase from *Thermobifida fusca* . Biochem Eng J 93: 222–228.

[mbt212714-bib-0005] Barth, M. , Wei, R. , Oeser, T. , Then, J. , Schmidt, J. , Wohlgemuth, F. , and Zimmermann, W. (2015b) Enzymatic hydrolysis of polyethylene terephthalate films in an ultrafiltration membrane reactor. J Membrane Sci 494: 182–187.

[mbt212714-bib-0006] Barth, M. , Honak, A. , Oeser, T. , Wei, R. , Belisário‐Ferrari, M.R. , Then, J. , *et al* (2016) A dual enzyme system composed of a polyester hydrolase and a carboxylesterase enhances the biocatalytic degradation of polyethylene terephthalate films. Biotechnol J 11: 1082–1087. 27214855 10.1002/biot.201600008

[mbt212714-bib-0007] Billig, S. , Oeser, T. , Birkemeyer, C. , and Zimmermann, W. (2010) Hydrolysis of cyclic poly(ethylene terephthalate) trimers by a carboxylesterase from *Thermobifida fusca* KW3. Appl Microbiol Biotechnol 87: 1753–1764. 20467738 10.1007/s00253-010-2635-y

[mbt212714-bib-0008] Carniel, A. , Valoni, É. , Nicomedes Junior, J. , Gomes, A.d.C. and Castro, A.M.d. (2016) Lipase from *Candida antarctica* (CALB) and cutinase from *Humicola insolens* act synergistically for PET hydrolysis to terephthalic acid. Process Biochem doi: 10.1016/j.procbio.2016.07.023.

[mbt212714-bib-0009] Chen, S. , Su, L. , Chen, J. , and Wu, J. (2013) Cutinase: characteristics, preparation, and application. Biotechnol Adv 31: 1754–1767. 24055682 10.1016/j.biotechadv.2013.09.005

[mbt212714-bib-0010] Chen, L. , Pelton, R.E.O. , and Smith, T.M. (2016) Comparative life cycle assessment of fossil and bio‐based polyethylene terephthalate (PET) bottles. J Cleaner Production 137: 667–676.

[mbt212714-bib-0011] Collias, D.I. , Harris, A.M. , Nagpal, V. , Cottrell, I.W. , and Schultheis, M.W. (2014) Biobased terephthalic acid technologies: a literature review. Ind Biotechnol 10: 91–105.

[mbt212714-bib-0012] Ellen MacArthur Foundation and World Economic Forum (2014) The New Plastics Economy ‐ Rethinking the future of plastics. URL https://www.ellenmacarthurfoundation.org/publications/the-new-plastics-economy-rethinking-the-future-of-plastics

[mbt212714-bib-0013] Fiber Economics Bureau (2015) 2015 World Directory of Manufactured Fiber Producers. 22 ed. URL http://www.fibersource.com/f-info/FiberProduction.pdf

[mbt212714-bib-0014] Gallagher, F.G. (2003) Controlled degradation polyesters In Modern Polyesters: Chemistry and Technology of Polyesters and Copolyesters. Scheirs J. and Long T.E. (eds). Chichester, UK: John Wiley & Sons, Ltd, pp. 591–608.

[mbt212714-bib-0015] Gamerith, C. , Zartl, B. , Pellis, A. , Guillamot, F. , Marty, A. , Acero, E.H. , and Guebitz, G.M. (2017) Enzymatic recovery of polyester building blocks from polymer blends. Proc Biochem. doi:10.1016/j.procbio.2017.01.004.

[mbt212714-bib-0016] Heredia, A. (2003) Biophysical and biochemical characteristics of cutin, a plant barrier biopolymer. Biochim Biophys Acta 1620: 1–7. 12595066 10.1016/s0304-4165(02)00510-x

[mbt212714-bib-0017] Herrero Acero, E. , Ribitsch, D. , Steinkellner, G. , Gruber, K. , Greimel, K. , Eiteljoerg, I. , *et al* (2011) Enzymatic surface hydrolysis of PET: effect of structural diversity on kinetic properties of cutinases from *Thermobifida* . Macromolecules 44: 4632–4640.

[mbt212714-bib-0018] Hunsen, M. , Abul, A. , Xie, W. , and Gross, R.A. (2008a) *Humicola insolens* cutinase‐catalyzed lactone ring‐opening polymerizations: kinetic and mechanistic studies. Biomacromol 9: 518–522. 10.1021/bm701269p 18198834

[mbt212714-bib-0019] Hunsen, M. , Azim, A. , Mang, H. , Wallner Sabine, R. , Ronkvist, A. , Xie, W. , and Gross, R.A. (2008b) Cutinase: a powerful biocatalyst for polyester synthesis by polycondensation of diols and diacids and ROP of lactones Polymer Biocatalysis and Biomaterials II. Cheng H.N. and Gross R.A. (eds). Washington, DC, USA: American Chemical Society, pp. 263–274.

[mbt212714-bib-0020] Jambeck, J.R. , Geyer, R. , Wilcox, C. , Siegler, T.R. , Perryman, M. , Andrady, A. , *et al* (2015) Plastic waste inputs from land into the ocean. Science 347: 768–771. 25678662 10.1126/science.1260352

[mbt212714-bib-0021] Kawai, F. , Oda, M. , Tamashiro, T. , Waku, T. , Tanaka, N. , Yamamoto, M. , *et al* (2014) A novel Ca^2+^‐activated, thermostabilized polyesterase capable of hydrolyzing polyethylene terephthalate from *Saccharomonospora viridis* AHK190. Appl Microbiol Biotechnol 98: 10053–10064. 24929560 10.1007/s00253-014-5860-y

[mbt212714-bib-0022] Kitadokoro, K. , Thumarat, U. , Nakamura, R. , Nishimura, K. , Karatani, H. , Suzuki, H. , and Kawai, F. (2012) Crystal structure of cutinase Est119 from *Thermobifida alba* AHK119 that can degrade modified polyethylene terephthalate at 1.76 Å resolution. Polym Degrad Stabil 97: 771–775.

[mbt212714-bib-0023] Kolattukudy, P.E. (1981) Structure, biosynthesis, and biodegradation of cutin and suberin. Annu Rev Plant Phys 32: 539–567.

[mbt212714-bib-0024] Manju , Kumar Roy, P. , Ramanan, A. and Rajagopal, C. (2013) Post consumer PET waste as potential feedstock for metal organic frameworks. Mater Lett 106, 390–392.

[mbt212714-bib-0025] Marten, E. , Müller, R.‐J. , and Deckwer, W.‐D. (2003) Studies on the enzymatic hydrolysis of polyesters I. Low molecular mass model esters and aliphatic polyesters. Polym Degrad Stabil 80: 485–501.

[mbt212714-bib-0026] Marten, E. , Müller, R.‐J. , and Deckwer, W.‐D. (2005) Studies on the enzymatic hydrolysis of polyesters. II. Aliphatic‐aromatic copolyesters. Polym Degrad Stabil 88: 371–381.

[mbt212714-bib-0027] Müller, R.‐J. (2006) Biological degradation of synthetic polyesters–Enzymes as potential catalysts for polyester recycling. Process Biochem 41: 2124–2128.

[mbt212714-bib-0028] Müller, R.‐J. , Schrader, H. , Profe, J. , Dresler, K. , and Deckwer, W.‐D. (2005) Enzymatic degradation of poly(ethylene terephthalate): rapid hydrolyse using a hydrolase from *T. fusca* . Macromol Rapid Comm 26: 1400–1405.

[mbt212714-bib-0029] Oeser, T. , Wei, R. , Baumgarten, T. , Billig, S. , Foellner, C. , and Zimmermann, W. (2010) High level expression of a hydrophobic poly(ethylene terephthalate)‐hydrolyzing carboxylesterase from *Thermobifida fusca* KW3 in *Escherichia coli* BL21(DE3). J Biotechnol 146: 100–104. 20156495 10.1016/j.jbiotec.2010.02.006

[mbt212714-bib-0030] Pellis, A. , Ferrario, V. , Zartl, B. , Brandauer, M. , Gamerith, C. , Herrero Acero, E. , *et al* (2016a) Enlarging the tools for efficient enzymatic polycondensation: structural and catalytic features of cutinase 1 from Thermobifida cellulosilytica. Catal Sci Technol 6: 3430–3442.

[mbt212714-bib-0031] Pellis, A. , Gamerith, C. , Ghazaryan, G. , Ortner, A. , Herrero Acero, E. , and Guebitz, G.M. (2016b) Ultrasound‐enhanced enzymatic hydrolysis of poly(ethylene terephthalate). Bioresource Technol 218: 1298–1302. 10.1016/j.biortech.2016.07.106 27481467

[mbt212714-bib-0032] Plastic Insight (2016) Global PET Resin Production Capacity. URL https://www.plasticsinsight.com/global-pet-resin-production-capacity/

[mbt212714-bib-0033] PlasticsEurope (2016) Plastics – the Facts 2016. URL http://www.plasticseurope.de/Document/plastics-the-facts-2016-15787.aspx

[mbt212714-bib-0034] Ribitsch, D. , Yebra, A.O. , Zitzenbacher, S. , Wu, J. , Nowitsch, S. , Steinkellner, G. , *et al* (2013) Fusion of binding domains to *Thermobifida cellulosilytica* cutinase to tune sorption characteristics and enhancing PET hydrolysis. Biomacromol 14: 1769–1776. 10.1021/bm400140u 23718548

[mbt212714-bib-0035] Ribitsch, D. , Herrero Acero, E. , Przylucka, A. , Zitzenbacher, S. , Marold, A. , Gamerith, C. , *et al* (2015) Enhanced cutinase‐catalyzed hydrolysis of polyethylene terephthalate by covalent fusion to hydrophobins. Appl Environ Microbiol 81: 3586–3592. 25795674 10.1128/AEM.04111-14 PMC4421044

[mbt212714-bib-0036] Ronkvist, Ã.S.M. , Xie, W. , Lu, W. and Gross, R.A. (2009) Cutinase‐catalyzed hydrolysis of poly(ethylene terephthalate). Macromolecules 42, 5128–5138.

[mbt212714-bib-0037] Roth, C. , Wei, R. , Oeser, T. , Then, J. , Foellner, C. , Zimmermann, W. , and Sträter, N. (2014) Structural and functional studies on a thermostable polyethylene terephthalate degrading hydrolase from *Thermobifida fusc* . Appl Microbiol Biotechnol 98: 7815–7823. 24728714 10.1007/s00253-014-5672-0

[mbt212714-bib-0038] Schmidt, J. , Wei, R. , Oeser, T. , Belisário‐Ferrari, M.R. , Barth, M. , Then, J. , and Zimmermann, W. (2016) Effect of Tris, MOPS, and phosphate buffers on the hydrolysis of polyethylene terephthalate films by polyester hydrolases. FEBS Open Bio 6: 919–927. 10.1002/2211-5463.12097 PMC5011490 27642555

[mbt212714-bib-0039] Smith, P.B. (2015) Bio‐based sources for terephthalic acid Green Polymer Chemistry: Biobased Materials and Biocatalysis. Cheng H.N., Gross R.A. and Smith P.B. (eds). Washington, DC, USA: American Chemical Society, pp. 453–469.

[mbt212714-bib-0040] Sulaiman, S. , Yamato, S. , Kanaya, E. , Kim, J.J. , Koga, Y. , Takano, K. , and Kanaya, S. (2012) Isolation of a novel cutinase homolog with polyethylene terephthalate‐degrading activity from leaf‐branch compost by using a metagenomic approach. Appl Environ Microbiol 78: 1556–1562. 22194294 10.1128/AEM.06725-11 PMC3294458

[mbt212714-bib-0041] Sulaiman, S. , You, D.J. , Kanaya, E. , Koga, Y. , and Kanaya, S. (2014) Crystal structure and thermodynamic and kinetic stability of metagenome‐derived LC‐cutinase. Biochemistry 53: 1858–1869. 24593046 10.1021/bi401561p

[mbt212714-bib-0042] Then, J. , Wei, R. , Oeser, T. , Barth, M. , Belisário‐Ferrari, M.R. , Schmidt, J. , and Zimmermann, W. (2015) Ca^2+^ and Mg^2+^ binding site engineering increases the degradation of polyethylene terephthalate films by polyester hydrolases from *Thermobifida fusca* . Biotechnol J 10: 592–598. 25545638 10.1002/biot.201400620

[mbt212714-bib-0043] Then, J. , Wei, R. , Oeser, T. , Gerdts, A. , Schmidt, J. , Barth, M. , and Zimmermann, W. (2016) A disulfide bridge in the calcium binding site of a polyester hydrolase increases its thermal stability and activity against polyethylene terephthalate. FEBS Open Bio 6: 425–432. 10.1002/2211-5463.12053 PMC4856421 27419048

[mbt212714-bib-0044] Thumarat, U. , Nakamura, R. , Kawabata, T. , Suzuki, H. , and Kawai, F. (2012) Biochemical and genetic analysis of a cutinase‐type polyesterase from a thermophilic *Thermobifida alba* AHK119. Appl Microbiol Biotechnol 95(2): 419–430. 22183084 10.1007/s00253-011-3781-6

[mbt212714-bib-0045] Tsiropoulos, I. , Faaij, A.P.C. , Lundquist, L. , Schenker, U. , Briois, J.F. , and Patel, M.K. (2015) Life cycle impact assessment of bio‐based plastics from sugarcane ethanol. J Cleaner Production 90: 114–127.

[mbt212714-bib-0046] Vertommen, M.A. , Nierstrasz, V.A. , Veer, M. , and Warmoeskerken, M.M. (2005) Enzymatic surface modification of poly(ethylene terephthalate). J Biotechnol 120: 376–386. 16115695 10.1016/j.jbiotec.2005.06.015

[mbt212714-bib-0047] Webb, H. , Arnott, J. , Crawford, R. , and Ivanova, E. (2013) Plastic degradation and its environmental implications with special reference to poly(ethylene terephthalate). Polymers 5: 1.

[mbt212714-bib-0048] Wei, R. and Zimmermann, W. (2017) Microbial enzymes for the recycling of recalcitrant petroleum‐based plastics: how far are we? Microb Biotechnol doi: 10.1111/1751‐7915.12710. 10.1111/1751-7915.12710 PMC5658625 28371373

[mbt212714-bib-0049] Wei, R. , Oeser, T. , Billig, S. , and Zimmermann, W. (2012) A high‐throughput assay for enzymatic polyester hydrolysis activity by fluorimetric detection. Biotechnol J 7: 1517–1521. 22623363 10.1002/biot.201200119

[mbt212714-bib-0050] Wei, R. , Oeser, T. , Barth, M. , Weigl, N. , Lübs, A. , Schulz‐Siegmund, M. , *et al* (2014a) Turbidimetric analysis of the enzymatic hydrolysis of polyethylene terephthalate nanoparticles. J Mol Catal B‐Enzym 103: 72–78.

[mbt212714-bib-0051] Wei, R. , Oeser, T. , Then, J. , Kühn, N. , Barth, M. , and Zimmermann, W. (2014b) Functional characterization and structural modeling of synthetic polyester‐degrading hydrolases from *Thermomonospora curvata* . AMB Express 4: 44. 25405080 10.1186/s13568-014-0044-9 PMC4231364

[mbt212714-bib-0052] Wei, R. , Oeser, T. , and Zimmermann, W. (2014c) Synthetic polyester‐hydrolyzing enzymes from thermophilic actinomycetes. Adv Appl Microbiol 89: 267–305. 25131405 10.1016/B978-0-12-800259-9.00007-X

[mbt212714-bib-0053] Wei, R. , Oeser, T. , Schmidt, J. , Meier, R. , Barth, M. , Then, J. , and Zimmermann, W. (2016) Engineered bacterial polyester hydrolases efficiently degrade polyethylene terephthalate due to relieved product inhibition. Biotechnol Bioeng 113: 1658–1665. 26804057 10.1002/bit.25941

[mbt212714-bib-0054] Wierckx, N. , Prieto, M.A. , Pomposiello, P. , de Lorenzo, V. , O'Connor, K. , and Blank, L.M. (2015) Plastic waste as a novel substrate for industrial biotechnology. Microb Biotechnol 8: 900–903. 26482561 10.1111/1751-7915.12312 PMC4621443

[mbt212714-bib-0055] Yoshida, S. , Hiraga, K. , Takehana, T. , Taniguchi, I. , Yamaji, H. , Maeda, Y. , *et al* (2016) A bacterium that degrades and assimilates poly(ethylene terephthalate). Science 351: 1196–1199. 26965627 10.1126/science.aad6359

[mbt212714-bib-0056] Zimmermann, W. , and Billig, S. (2011) Enzymes for the biofunctionalization of poly(ethylene terephthalate). Adv Biochem Eng Biotechnol 125: 97–120. 21076908 10.1007/10_2010_87

